# A new quantitative evaluation system for distal radioulnar joint instability using a three-dimensional electromagnetic sensor

**DOI:** 10.1186/s13018-021-02601-4

**Published:** 2021-07-14

**Authors:** Shintaro Mukohara, Yutaka Mifune, Atsuyuki Inui, Hanako Nishimoto, Takashi Kurosawa, Kohei Yamaura, Tomoya Yoshikawa, Issei Shinohara, Yuichi Hoshino, Kouki Nagamune, Ryosuke Kuroda

**Affiliations:** 1 Department of Orthopaedic Surgery, Kobe University Graduate School of Medicine, Kobe, Japan; 2 Department of Human and Artificial Intelligent Systems, School of Engineering, University of Fukui, Fukui, Japan

**Keywords:** Distal radioulnar joint, Instability, Dynamic instability, Electromagnetic sensor system, Triangular fibrocartilage complex injury, TFCC injury

## Abstract

**Background:**

The accurate assessment of distal radioulnar joint (DRUJ) instability is still challenging as there is no established objective evaluation method. This study aimed to develop a noninvasive measurement method using a three-dimensional electromagnetic sensor system (EMS) to quantitatively assess and characterize the normal DRUJ movement in healthy volunteers.

**Methods:**

The DRUJ movement was mimicked using both a block model and saw bone. Movement of the models was measured by EMS, and the accuracy and reproducibility of the measurements were assessed. In vivo measurement was performed in a sitting position with the elbow flexed and the forearm pronated. One sensor each was attached to the distal radial shaft and the ulnar head. The examiners fixed the distal radius and the carpal bones, moved the ulnar head from the dorsal to the volar side and measured the dorsovolar translation. The volar translation was measured by EMS and ultrasonography, and the correlation coefficient was calculated. The dorsovolar translation was evaluated in 14 healthy volunteers (7 men and 7 women) by three hand surgeons. The intraclass and inter-rater correlation coefficients (ICCs), the differences between the dominant and non-dominant sides and between men and women were assessed.

**Results:**

The accuracy and reproducibility assessment results of the EMS showed high accuracy and reproducibility. In the comparison between EMS and ultrasonography, the correlation coefficient was 0.920 (*p* = 0.16 × 10^-3^). The ICC (1,5) for the intra-rater reliability was 0.856, and the ICC (2,5) for inter-rater reliability was 0.868. The mean ulnar head translation and difference between dominant and non-dominant sides were 6.00 ± 1.16 mm (mean ± SD) and − 0.12 ± 0.40 mm, respectively. There were no significant differences between any of the parameters.

**Conclusions:**

A new measurement method using EMS could evaluate DRUJ movement with high accuracy, reproducibility, and intra- and inter-rater reliability. In healthy volunteers, the dorsovolar ulnar head translation was 6.00 mm. The difference between the dominant and non-dominant sides was < 1.0 mm with no significant difference. EMS provided an objective, non-invasive, real-time assessment of dynamic changes in the DRUJ. These findings could be useful in the treatment of patients with DRUJ instability.

## Background

The stability of the distal radioulnar joint (DRUJ) is provided by the contour of the bones as well as the surrounding ligaments and muscles, such as the triangular fibrocartilage complex (TFCC), ulnocarpal ligament complex, extensor carpi ulnaris tendon and tendon sheath, the pronator quadratus muscle, the interosseous membrane including the interosseous ligament, and the capsule [1–5]. The TFCC particularly contributes to its stability, and TFCC injuries cause instability of the DRUJ, leading to chronic ulnar wrist pain [6, 7]. Various manual tests for assessing DRUJ instability have been reported, such as the ballottement test, Piano key test, and Pisiform boot test. Some reports suggest that the ballottement test is the most reliable [8]; however, these manual tests depend on subjective evaluations. It is difficult to accurately assess instability in clinical practice, and there is no well-established method to objectively evaluate DRUJ instability.

Recently, a knee motion quantitative assessment method with high reproducibility using a three-dimensional electromagnetic sensor system (EMS) was reported [9–16]. This system can quantitatively evaluate knee laxity after anterior cruciate ligament (ACL) injury with a high sampling rate during the Lachman test and the pivot shift test, which have been used as manual examination methods for detecting ACL deficiency [9–16]. We hypothesized that EMS could be used to quantitatively evaluate DRUJ instability. Furthermore, it is important to establish a normal range to define DRUJ instability. Thus, the purpose of this study was to develop a new objective evaluation method for DRUJ instability using EMS, and to quantitatively evaluate DRUJ movements in healthy volunteers.

## Methods

### Electromagnetic sensor system (EMS)

**Fig. 1:**
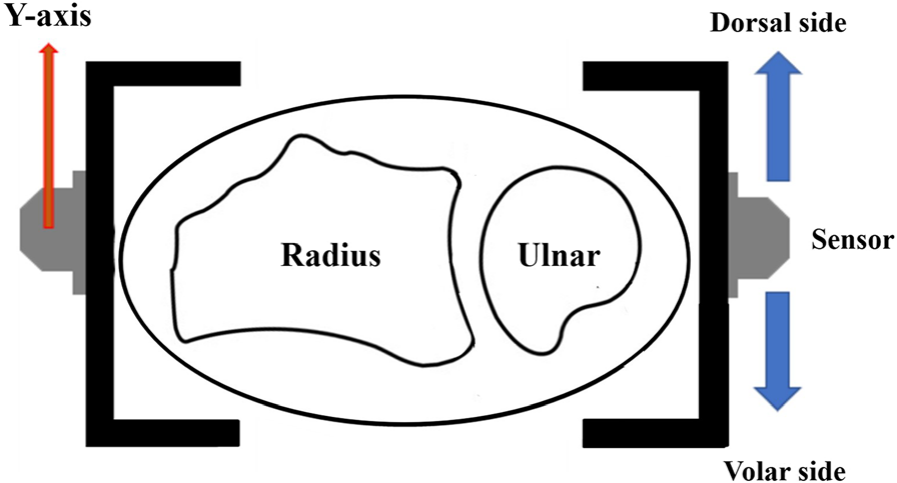
Electromagnetic measurement system

All experimental measurements were performed using an electromagnetic device (Liberty®, Polhemus, VT, USA). The system consists of a transmitter that produces an electromagnetic field and two three-dimensional electromagnetic sensors. This system had a root mean square accuracy of 0.76 mm for position and 0.15° for orientation when it was used within an optimal operational zone with transmitter-to-sensor separation within 106 cm, and there was no interference from the magnetic material [17]. Two sensors were used for motion measurement and attached to the radial aspect of the distal radius shaft (10 cm proximal to the radial styloid process) and the ulnar aspect of the ulnar head, respectively. The ulnar head translation with reference to the Y-axis of the sensor on the radial side was calculated on a personal computer using coordinated software (Fig. 1). This system could measure with high sample rates (60Hz) and measurements were reflected on the monitor of personal computer in real time.

### Assessment of EMS accuracy

**Fig. 2:**
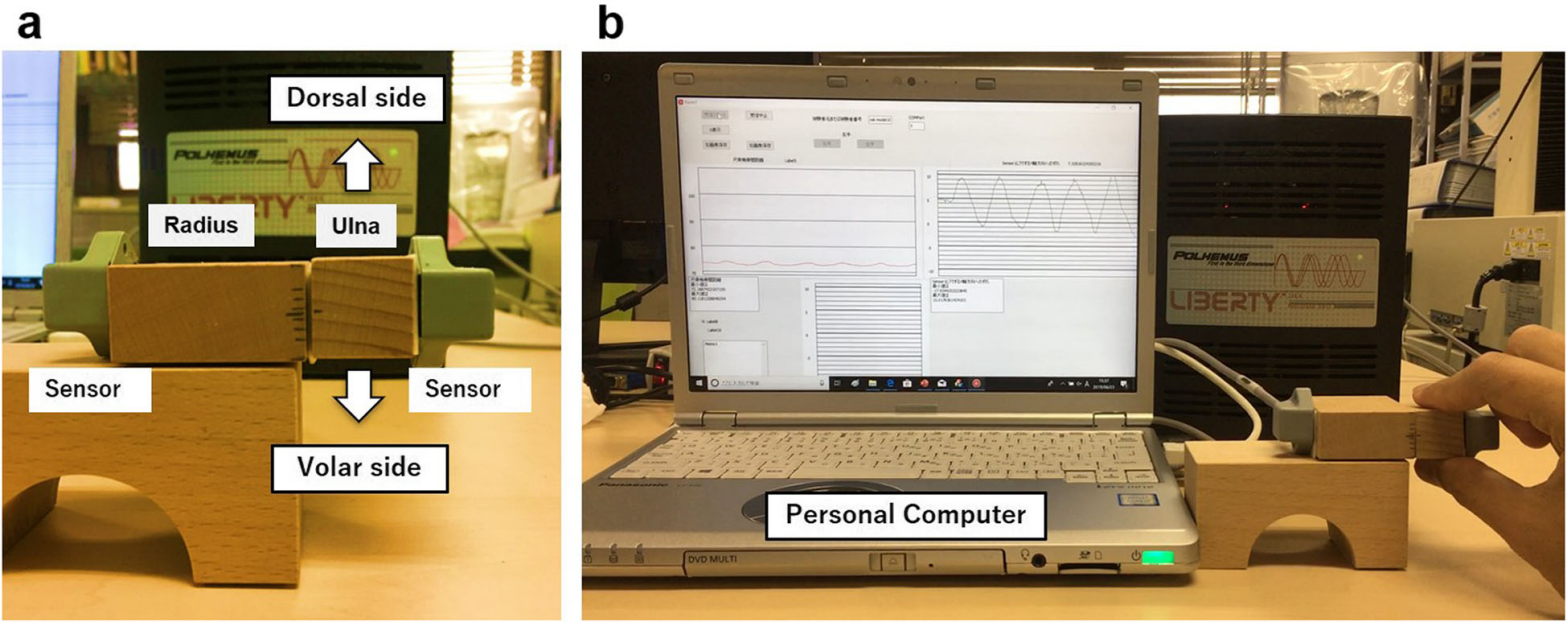
**a** Each electromagnetic sensor was attached to the blocks which imitated the radius and ulna of DRUJ in forearm pronation. The ulnar block was moved to volar side and dorsal side. b The measurements can be monitored in real time on a screen of personal computer

Each sensor was attached to the blocks which imitated the radius and ulna of the DRUJ in forearm pronation (Fig. 2a, b). Landmarks of 1, 3, 5, and 10 mm were created on the radial side based on a scale that can measure down to 0.1 mm. One examiner manually moved the ulnar block to dorsal and volar sides by 1, 3, 5, and 10 mm, respectively. Measurements were performed seven times on each side. The maximum and minimum values were excluded as outliers, and the mean value of the remaining five measurements was used for the analysis. Accuracy was assessed by calculating the error between the mean value of the measurements and the true value, standard deviation (SD), and Pearson’s correlation coefficient.

### Assessment of EMS reproducibility

**Fig. 3:**
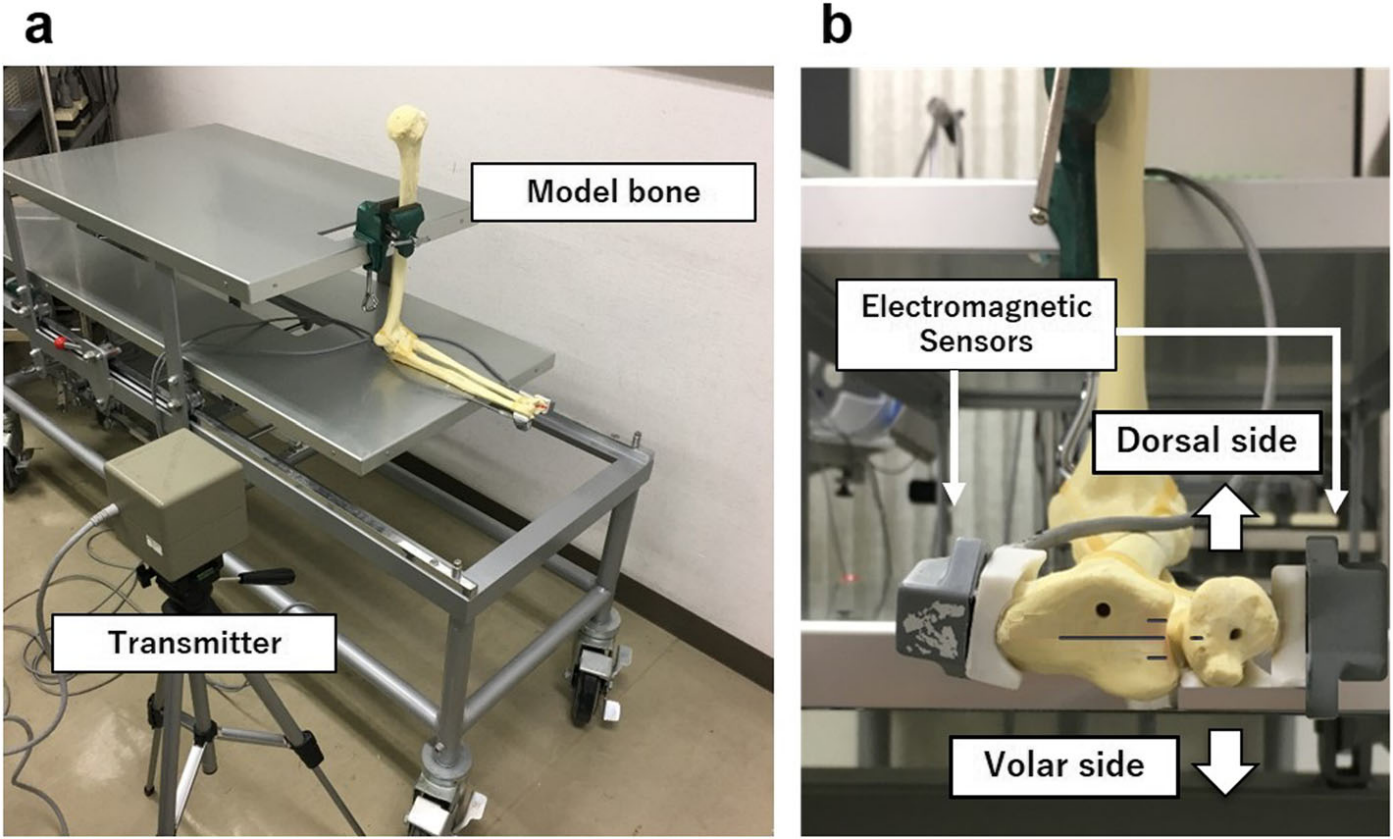
**a** The bone model of the upper limb was fixed in 90° elbow flexion, and the radius was in pronation. **b** Two sensors were attached to the radius and the ulnar styloid process

The bone model of the upper limb was fixed in 90° elbow flexion, and the radius was in pronation (Fig. 3a). Two sensors were attached to the radial aspect of the distal radial shaft and ulnar aspect of the ulnar head, respectively (Fig. 3b). Landmarks were made on the radial side at 5 mm each on the dorsal and volar sides, based on a scale that measures down to 0.1 mm. The examiner manually moved the ulnar head from the dorsal to the volar side for a total 10 mm, mimicking the ballottement test, and measured the ulnar head translation relative to the radius. Measurements were performed seven times by five examiners, including the removal and attachment of sensors. The maximum and minimum values were excluded as outliers, and the mean value of the remaining five measurements was used for the analysis. Reproducibility was assessed to calculate the SD.

### In vivo measurement

The protocol for in vivo measurements was reviewed and approved by our Institutional Review Board (No. B210009).

### Measurement technique using EMS

**Fig. 4:**
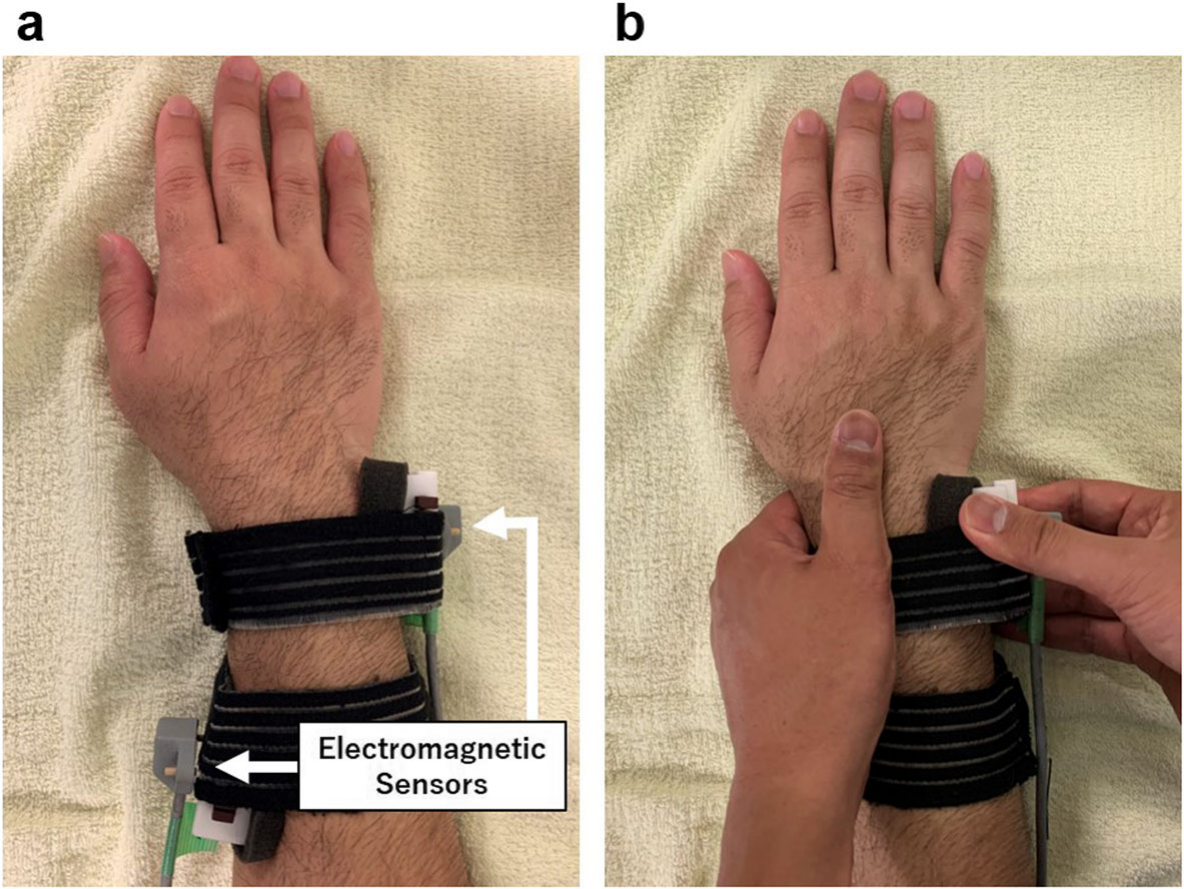
**a** Measurements were taken with the elbow flexed to 90° and the forearm in pronation. Each sensor was fixed to the side surface of jigs that could grip the distal radial shaft and the ulnar head. **b** The examiners grasped and fixed the distal end of the radius and carpal bones. The ulnar head was moved from the dorsal to the volar sides to measure the dorsovolar ulnar head translation with respect to the radius

Measurements were performed in the sitting position with the elbow flexed and the forearm 90° pronated. This limb measurement position, which mimics the piano key test, is highly reproducible in clinical practice. Each sensor was fixed to the side surface of the jigs that could grip the distal radial shaft (10 cm proximal to the radial styloid process) and the ulnar head from the dorsal and volar sides (Fig. 4a). The sensor on the radial side was placed slightly proximal to the DRUJ to prevent the influence of manual examination technique and ulnar head movement. The examiners grasped and fixed the distal end of the radius and the carpal bones while moving the ulnar head from the dorsal to the volar side, mimicking the holding technique of ballottement test [18], to measure the dorsovolar ulnar head translation with respect to the radius (Fig. 4b). The examiner moved the ulnar head 10 times in one measurement. The first and last two times were excluded, and the mean value of the remaining six times was taken as the result of one measurement. Measurements were taken seven times per subject. The maximum and minimum values were excluded as outliers, and the mean of remaining five measurements were used for the analysis.

### Comparison of EMS and ultrasonography

**Fig. 5:**
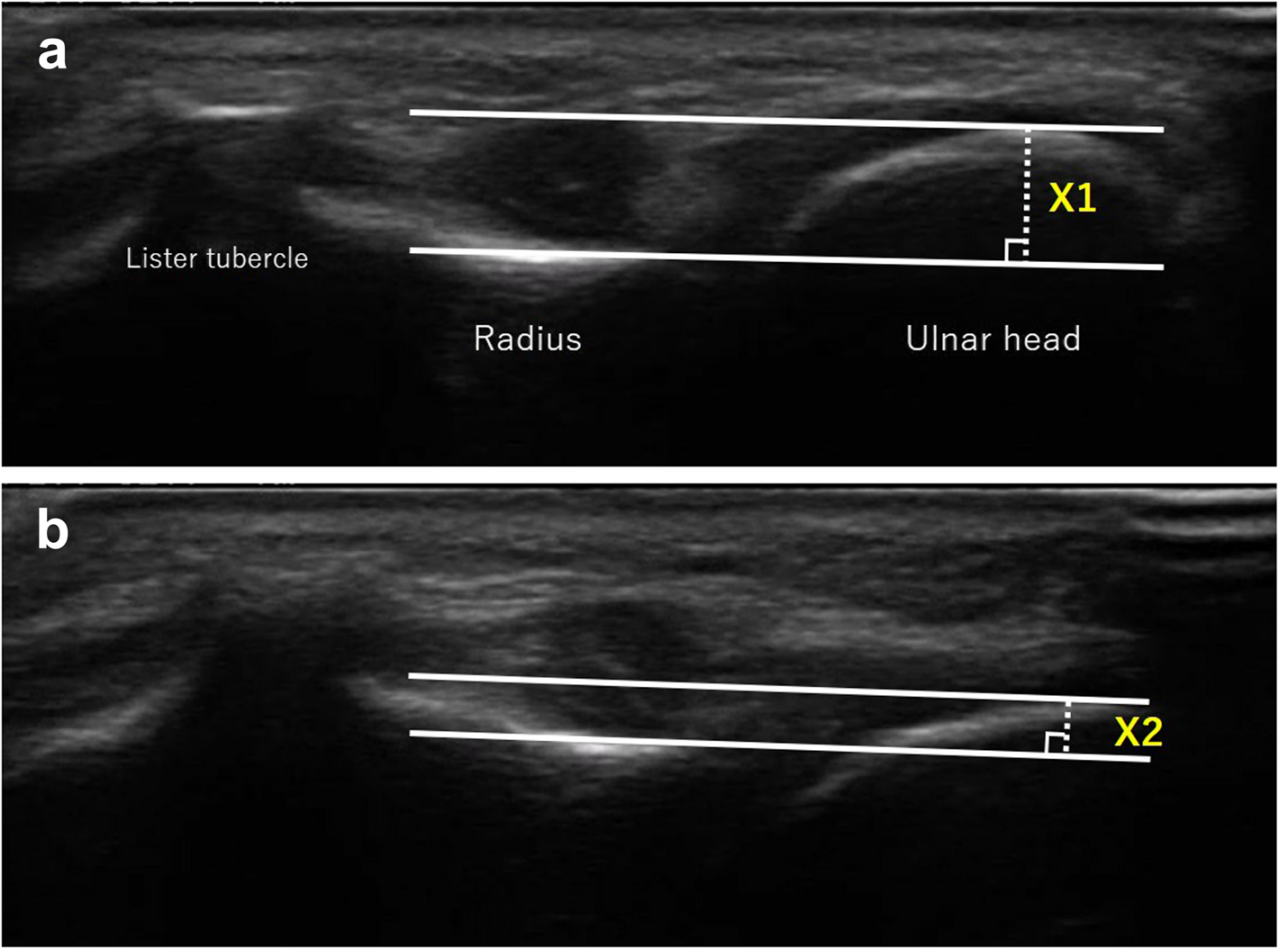
The ultrasonography of the distal radioulnar joint. **a** Before the compression of the ulnar head. **b** After the compression of the ulnar head. X1: distance between the dorsal surfaces of the ulnar head and the radius before compression. X2: distance between the dorsal surfaces of the ulnar head and the radius after compression. X1-X2: the volar translation distance of the ulnar head

The experienced hand surgeon measured the dominant hand of 10 healthy volunteers (10 men), who had no history of wrist trauma or pain, with a mean age (and SD) of 34.6 ± 6.7 years. Measurements using EMS and calculations of measurement results were performed in the manner described above, but the ulnar head was moved to only the volar side for comparison with ultrasonography. Measurements using an ultrasound system (Prologue, Hitachi Aloka Medical, Ltd., Tokyo, Japan) were performed in the same position. According to the previous report [19], the transducer was placed dorsally above the DRUJ, perpendicular to the longitudinal axis of the ulna. The dorsal surface of the distal radius and the center of the ulnar head were displayed on a monitor. To determine the same measurement level in each volunteer, the highest aspect of the ulnar head was taken. With the transducer fixed in that position, the examiner held the distal radius and compressed the ulnar head to the volar side five times. The distance between the dorsal aspect of the radius and the dorsal aspect of the ulnar head was measured before (X1) and after the volar compression of the ulnar head (X2), and the difference between the two measurements (= X1 − X2, mm) was defined as the translation distance to volar side of the ulnar head (Fig. 5). The first and last two times were excluded, and the mean value of the remaining three times was defined as the result of one measurement. Measurements were taken five times per subject. The maximum and minimum values were excluded as outliers, and the mean of remaining three measurements were used for the analysis. Pearson’s correlation coefficient between EMS and ultrasonography were calculated from the measurement results.

### Measurement of healthy volunteers using EMS

Three experienced hand surgeons measured the dominant and non-dominant hands of 14 healthy volunteers (7 men and 7 women) with a mean age (and SD) of 33.4 ± 5.9 years. Volunteers were excluded if they had a history of wrist trauma or pain. The intraclass correlation coefficient (ICC) was calculated using the mean value of the measurements, and the intra- and inter-rater reliabilities were evaluated. The differences between the dominant and non-dominant sides and between men and women were assessed.

### Statistical analysis

The results are expressed as mean ± SD. The Mann-Whitney *U* test was used for comparisons between the two groups. The level of significance was set at *p* < 0.05. Statistical analyses were performed using the Excel statistical software package (Ekuseru-Toukei 2015; Social Survey Research Information Co., Ltd., Tokyo, Japan) and SPSS Statistics (IBM, Tokyo, Japan) software.

## Results

### Assessment of EMS accuracy and reproducibility

**Fig. 6:**
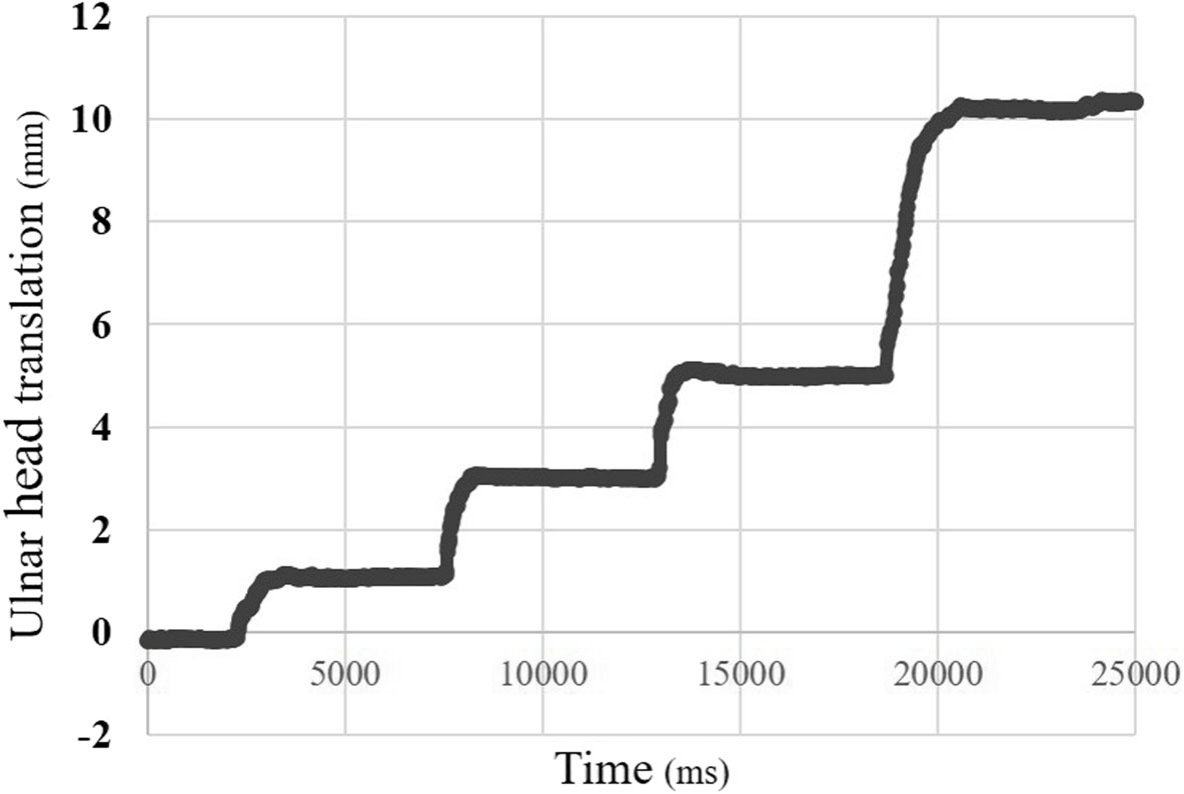
The ulnar block movement during measurement with a timeline

Figure 6 shows the accuracy assessment results of the ulnar block movement during the measurements with a timeline. The measurements are shown in Table 1. When the ulnar block was moved 1, 3, 5, and 10 mm to the volar side, the measurements were 1.03 ± 0.04, 2.90 ± 0.06, 4.99 ± 0.17, and 9.92 ± 0.17 mm, respectively. The error between the mean value of the measurements and the true value was less than 0.2 mm in all circumstances, and the SD was less than 0.1 mm. Pearson’s correlation coefficient was 0.997 (*p* = 0.89 × 10^-7^).

**Table 1:** The measurements of ulnar block movement for assessment of accuracy

		1	2	3	4	5	Average (mm)	SD(mm)
**Dorsal side (mm)**	10	9.98	9.7	9.98	9.78	10.19	9.92	0.17
	5	5.12	4.72	4.94	5.21	4.99	4.99	0.17
	3	2.95	2.79	2.89	2.91	2.99	2.9	0.06
	1	0.97	1.08	1.07	1.01	1.04	1.03	0.04
**Palmar side (mm)**	− 1	− 1.09	− 1.11	− 1.07	− 0.97	− 1.12	− 1.07	0.06
	− 3	− 3.26	− 3.14	− 3.24	− 3.11	− 3.14	− 3.18	0.06
	− 5	− 5.03	− 5.08	− 5.18	− 5.21	− 5.16	− 5.13	0.07
	− 10	− 10.13	− 10.29	− 10.16	− 10.1	− 10.03	− 10.14	0.09

**Fig. 7:**
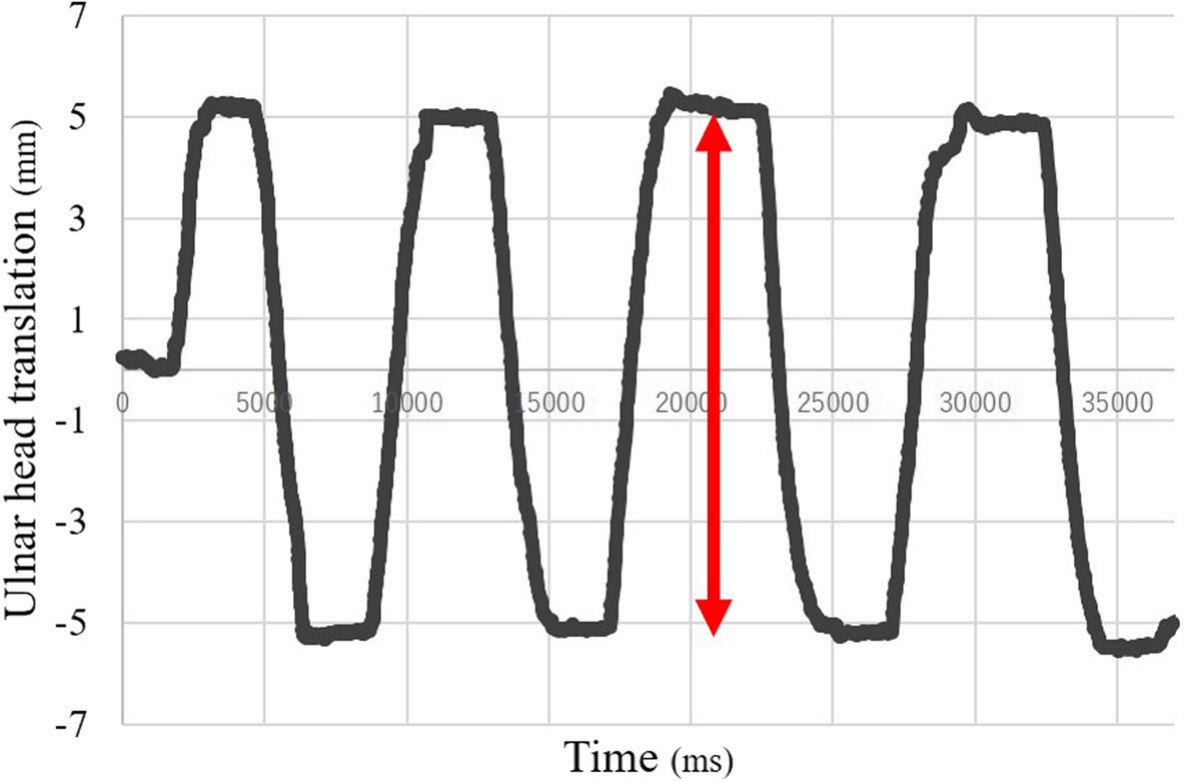
The ulnar head movement during measurement with a timeline

Figure 7 shows the reproducibility assessment of the ulnar head movement during the measurements with a timeline. The measurements are shown in Table 2. When five examiners moved the ulnar head by 10 mm, the mean ulnar head translation was 10.08 ± 0.17 mm.

**Table 2:** The measurements of ulnar head movement for assessment of reproducibility

	Examiner					Average (mm)	SD (mm)
	A	B	C	D	E		
Ulnar head translation (10 mm)	10.16	10.2	9.99	10.01	10.03	10.08	0.17

### In vivo measurement

#### Comparison of EMS and ultrasonography

**Fig. 8:**
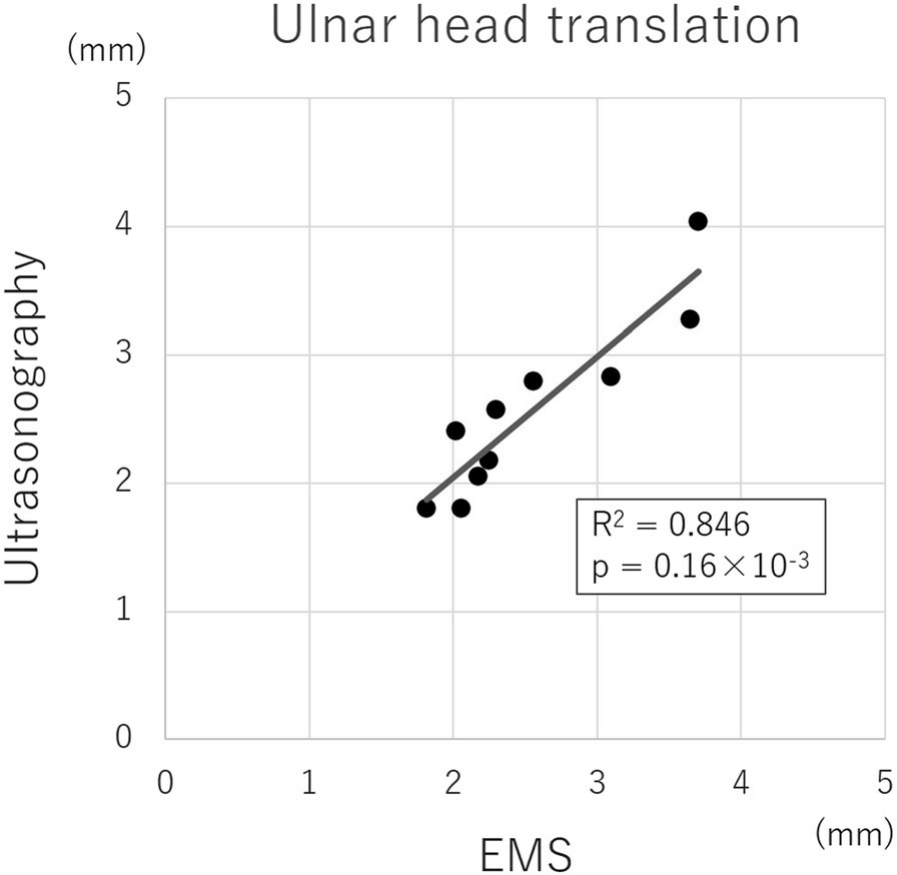
Relationship of the ulnar head translation between EMS and ultrasonography

The mean value of the volar translation of the ulnar head measured by EMS was 2.56 ± 0.64 mm (range, 1.82–3.71 mm). The mean value of the volar translation of the ulnar head measured by ultrasonography was 2.57 ± 0.66 mm (range, 1.80–4.04mm). Pearson’s correlation coefficient was 0.920 (*R*^2^ = 0.84, *p* = 0.16 × 10^-3^) (Fig. 8).

#### Measurement of healthy volunteers using EMS

**Fig. 9:**
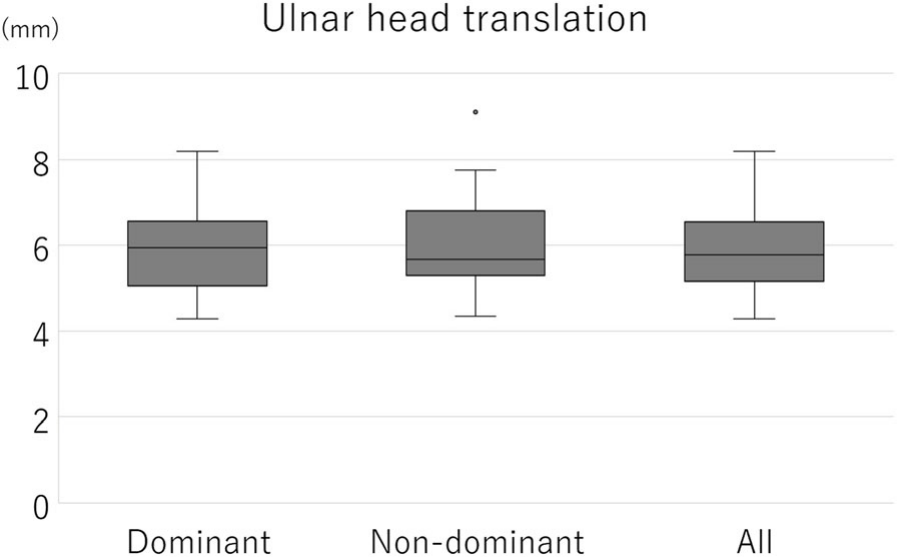
The ulnar head translation on dominant and non-dominant sides

**Fig. 10:**
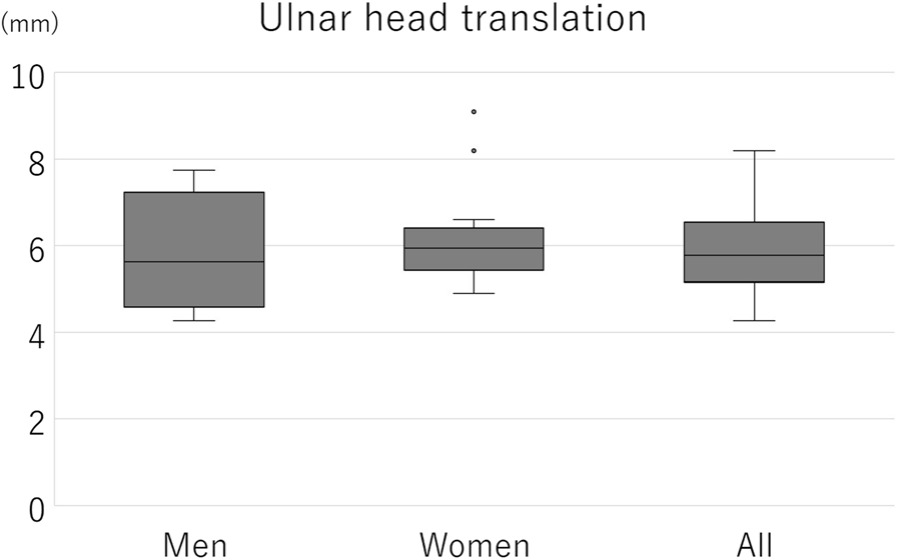
The ulnar head translation in men and women

**Fig. 11:**
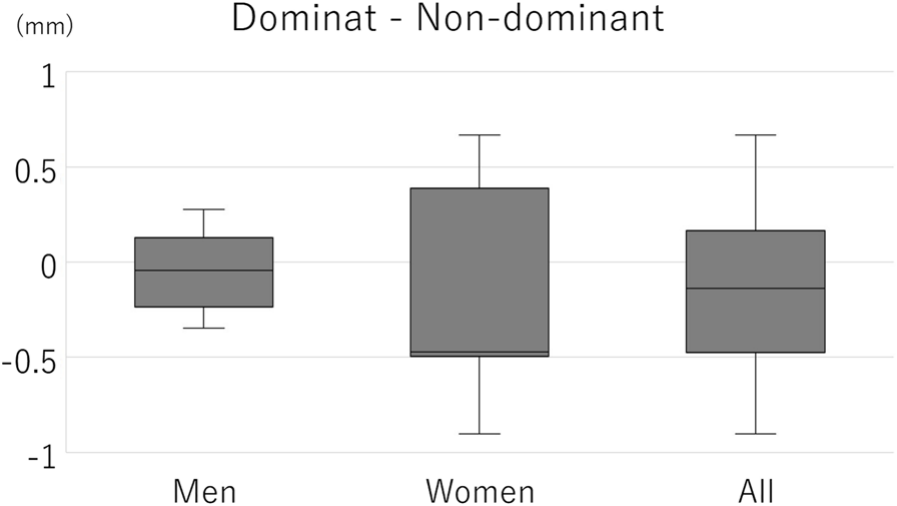
The difference in ulnar translation between the dominant and non-dominant sides in men and women

The ICC (1,5) indicating intra-rater reliability was 0.856, and the ICC (2,5) indicating inter-rater reliability was 0.868. The mean ulnar head translation of all measurements was 6.00 ± 1.16 mm (range, 4.27–9.10 mm) and that of the dominant and non-dominant sides were 5.93 ± 1.06 mm and 6.05 ± 1.25 mm, respectively (Fig. 9). There were no significant differences between the dominant and non-dominant sides. The mean ulnar head translation of men and women were 5.78 ± 1.18 mm and 6.22 ± 1.10 mm, respectively (Fig. 10). The mean of the difference between the dominant and non-dominant sides was − 0.12 ± 0.40 mm (−0.90 to 0.67 mm) and that of men and women were − 0.06 ± 0.21 mm and − 0.17 ± 0.52 mm, respectively (Fig. 11). There was no significant difference between men and women regarding the amount of ulnar head movement and the difference between the dominant and non-dominant sides.

## Discussion

Several methods using imaging data have been reported to objectively evaluate DRUJ instability, including plain radiography, computerized tomography (CT), and ultrasound examination [20–32]. Nakamura et al. reported that DRUJ subluxation and dislocation were indicated when the difference in the radioulnar distance between the affected and non-affected wrists was 6 mm or more on a normal lateral radiograph [20]. Additionally, on a posteroanterior radiograph, a widened gap between the distal radius and the ulna with respect to the unaffected side is a strong indicator of dorsal ulnar subluxation/dislocation, while increased overlap indicates volar ulnar subluxation/dislocation [21, 22]. However, a true lateral view of the DRUJ was difficult to take and, as little as 10° of supination or pronation, made radiographic diagnoses inaccurate [21–23].

Bilateral CT evaluation of the DRUJs is useful for detecting differences in anatomical details and DRUJ congruency between normal and injured wrists [24, 25]. There are various methods of quantifying the instability on axial CT images, such as the radioulnar line (or Mino’s) method, the radioulnar ratio method, the subluxation ratio method, the epicenter method, and the congruency method [21, 26–30]. The radioulnar line method and the congruency method showed high false-positive rates [31], while the epicenter method was the most specific and reliable among them [30, 31]. However, there was no clear statistical correlation between the stress test and CT parameters for DRUJ instability after distal radius fracture [32]. In addition, since both plain radiography and CT are static evaluations, the instability of DRUJ could be underestimated.

Recently, musculoskeletal evaluation using ultrasonography has become widespread. The potential advantages of ultrasound are its noninvasiveness, low cost, lack of ionizing radiation risk, and dynamic and real-time evaluation. Hess et al. reported a sonographic method of quantifying DRUJ instability by measuring volar ulnar head translation relative to the distal radius with the forearm pronated and distinguished a normal from an unstable DRUJ [19]. They showed that the average volar translation and differences between both wrists with normal DRUJ were 2.5 mm and 0.65 mm and those of unstable DRUJ were 5.8 mm and 2.8 mm [19]. However, they only assessed volar side instability and may have underestimated the DRUJ instability. In addition, ultrasound devices remain dependent on the operator and experience.

The reliability of EMS has been reported in the quantification of the Lachman test and pivot-shift test, which evaluate knee laxity after ACL injuries [9–16]. The measurements could be useful for understanding the pathophysiology of ACL injury pattern [9–16]. In this study, a new quantitative evaluation system for DRUJ movement using an EMS was developed. As a result of the accuracy assessment of the EMS, the error between the mean of the measurements and the true value was < 0.2 mm, SD was < 0.1 mm and Pearson’s correlation coefficient was 0.997, indicating high accuracy. Similarly, the reproducibility evaluation of the EMS showed that SD was < 0.17 mm, indicating high reproducibility. In in vivo measurements, the ulnar head translation to the volar side compared between EMS and ultrasonography was also significantly higher with Pearson’s correlation coefficient of 0.920 (*R*^2^ = 0.84, *p* = 0.16 × 10^-3^). Furthermore, in vivo measurements of the ICC demonstrated almost perfect intra- and inter-rater reliabilities [33], with an ICC (1,5) of 0.856 and an ICC (2,5) of 0.868. These results suggest that the EMS could be a clinically useful measurement method for quantifying DRUJ movement.

A previous cadaveric study investigated DRUJ movement during the ballottement test with a holding technique using a magnetic sensor system and reported that the average movement before TFCC sectioning was 9.8 mm [18]. In the in vivo measurements of this study, the mean dorsovolar ulnar head translation in healthy volunteers was approximately 6.0 mm, which was lower than that in the cadaveric report. This is thought to be due to the effect of dynamic stability caused by muscle contraction and the influence of using the holding technique in the forearm pronated position. Ultrasound measurements reported a volar ulnar head translation of 2.5 mm in the forearm pronated position [19]. In the present study, the mean of the ulnar head translation to volar side measured by EMS was 2.56 mm with similar results.

In the comparison between the dominant and non-dominant sides, ulnar head translation was slightly greater on the non-dominant side; however, there were individual differences, and no significant differences were found. Although the translation distance of females tended to be higher than that of males, there was no significant difference between males and females in each parameter, which was similar to a previous report using CT evaluation [34]. DRUJ instability varies greatly among individuals and is difficult to assess, especially in patients with joint laxity. This study also had a large normal range of 4.28–9.10 mm. Therefore, it is important to compare the difference between the healthy side and the affected side [29, 30, 34]. In the cadaveric study mentioned above, DRUJ instability increased by 2.3 mm after TFCC sectioning [18]. The ultrasonographic study reported that the average difference between both wrists with normal DRUJ was 0.65 mm, while with unstable DRUJ, it was 2.8 mm [19]. Another study on CT assessment under stress in the neutral position reported that a contralateral difference of 2–3 mm suggested instability [34]. In this study, the average difference between the dominant and non-dominant sides was 0.11 mm (0.01 to 0.90 mm) in healthy volunteers. These results suggest that a difference of < 1 mm between both wrists might be considered a stable DRUJ in the EMS measurement.

The advantage of the EMS is that dynamic changes in the DRUJ can be assessed objectively and in real time without any invasion or exposure [9–16]. TFCC injury is the main cause of DRUJ instability, but some cases are difficult to diagnose even with MRI or arthrography. Thus, objective measurement using EMS could help in the diagnosis and understanding of the pathology. Furthermore, EMS can be used for postoperative evaluation. These benefits demonstrate the potential of EMS as a clinically useful test.

This study has several limitations. First, the effect of the skin motion was not evaluated. Therefore, it would be preferable to assess this in a cadaveric study. However, the influence of skin motion was minimized by devising a measurement device to grip the DRUJ. Furthermore, our in vivo measurements showed that EMS correlated highly with ultrasonography, showed high intra- and inter-rater reliabilities, and the measurement results were also reasonable compared with that of the cadaveric study using the magnetic sensor system [18]. Second, the material used to fix the sensor differed between the blocks and bone models and in vivo measurement. However, to increase the fixation force of the sensor, the jig that could firmly grip the bone was created and the sensor was fixed on the jig, instead of gluing the sensor to the skin. In addition, the use of bands might reduce the DRUJ instability, but the tightening force was adjusted to a degree that would not affect the manual examination. Based on the results of comparison with ultrasonography, the differences in the material of sensor fixation and the effect of the band on the DRUJ instability were minimized. Thirdly, the sample size was small; however, we successfully confirmed the effectiveness of the measurement system and the values from healthy subjects in this study. We would like to further increase the sample size and compare the results with those of the patient group in the future.

## Conclusions

In this study, a new measurement method using an EMS was used to evaluate the movement of the DRUJ with high accuracy, reproducibility, and intra- and inter-rater reliabilities. In this measurement method, the dorsovolar ulnar head translation was approximately 6.00 mm, and the difference between the dominant and non-dominant sides was < 1.0 mm in healthy subjects. EMS can evaluate dynamic changes in the DRUJ objectively, non-invasively, and in real-time, suggesting that it could be a clinically applicable measurement method.

## Data Availability

The datasets used and/or analyzed during the current study are available from the corresponding author on reasonable request.

## Abbreviations

ACL: Anterior cruciate ligament
CT: Computed tomography
DRUJ: Distal radioulnar joint
EMS: Electromagnetic sensor system
ICC: Inter-rater/intraclass correlation coefficient
SD: Standard deviation
TFCC: Triangular fibrocartilage complex
